# Inhibition of Arterial Allograft Intimal Hyperplasia Using Recipient Dendritic Cells Pretreated with B7 Antisense Peptide

**DOI:** 10.1155/2012/892687

**Published:** 2012-02-06

**Authors:** Yu-Feng Yao, Yi-Ming Zhou, Jian-Bin Xiang, Xiao-Dong Gu, Duan Cai

**Affiliations:** ^1^Department of General Surgery, Huashan Hospital, Fudan University, Shanghai 200040, China; ^2^Department of General Surgery, Jiangsu Cancer Hospital and Cancer Research Institute, Nanjing 210009, China

## Abstract

*Background*. Low expression or absence of dendritic cell (DC) surface B7 molecules can induce immune tolerance or hyporesponse. Whether DCs could induce indirect allogeneic-specific cross-tolerance or hyporesponse to recipient T cells remains unclear. *Methods*. Generated from C3H/He mice bone marrow cells pulsed with donor antigen from C57BL/6 mice, recipient DCs were incubated with B7 antisense peptide (B7AP). Immune regulatory activities were examined *in vitro* by a series of mixed lymphocyte reactions. Murine allogeneic carotid artery orthotopic transplantation was performed from C57BL/6 to C3H/He. Recipients were given B7AP-treated DCs 7 days before transplantation. Allograft pathological analysis was done 2 months after transplantation. *Results*. B7AP-pretreated DCs markedly inhibited T-cell proliferation compared with untreated group. Pretreated T cells exhibited markedly reduced response to alloantigen versus third-party antigen. Pathological analysis of arterial allografts demonstrated significant reduction of intimal hyperplasia in B7-AP pretreated group versus control. *Conclusion*. Blockade of B7 molecules by B7AP could induce indirect allogeneic-specific hyporesponse and inhibit arterial allograft intimal hyperplasia, which may be involved in future strategies for human allograft chronic rejection.

## 1. Introduction

Graft loss from chronic rejection has become the major obstacle to the long-term success of whole organ transplantation [1]. It is accepted that direct and indirect recognitions, both mediated by donor and recipient dendritic cells (DCs), are the major causes for all types of organ transplant rejection [2, 3]. The direct activation of T lymphocytes by donor-derived antigen-presenting cells (APCs) is thought to be responsible for the vigor of acute rejection, while the indirect allorecognition has been implicated in the initiation of chronic allograft dysfunction [4–6].

The B7-CD28/CTLA4 costimulatory pathway plays a crucial role in the regulation of T-cell activation [7]. B7 molecules are expressed on the surface of APCs, providing a critical co-stimulatory signal to T cells by engaging CD28. Blockade of the B7-CD28 interaction *in vitro *can generate antigen-specific anergy [8–10]. Administration of monoclonal antibodies (MoAbs) against B7 or CTLA4-Ig fusion protein to block B7 has been shown to be promising as a treatment for allograft rejection [11–13]. It has been reported that the recipients immunized with donor resting B cells or immature DCs could induce specific immune tolerance and prolong allograft survival. This effect has been attributed to low-level expression or absence of B7 molecules on these cells [14, 15]. Although recent studies [16–18] have shown that modified or pretreated DCs can induce direct alloreactive T-cell hyporesponsiveness, it is not yet clear whether DCs can also induce indirect allogeneic-specific cross-hyporesponsiveness to recipient T cells. Recent years have witnessed an increasing interest in the development of nonimmunogenic peptide as an antagonist for protein-protein interaction in immunomodulatory therapeutics [19, 20]. Progress in antisense technology and molecular modeling over the past decade has made molecular recognition study possible [21–23]. Antisense peptides are short peptide sequences that specifically constitute one side of the binding sites of complementary protein pairs [24]. B7 antisense peptide (B7AP) is a peptide analogue of the CD28-binding region [12, 24]. It is characterized by higher affinity to B7 ligand, lower molecular weight, and lower immunogenicity and difficulty to be metabolized compared with CTLA4-Ig [12, 25] which would block the allorecognition in a longer period to take more effect. It has been reported [12] that blockade of B7 molecules on donor splenocytes pretreated with B7AP could induce specific immune hyporesponse and prolong allograft survival in the recipients. In this study, we tried to induce cross-hyporesponsiveness to recipient T cells in an indirect pathway by B7AP pretreated donor-pulsed recipient DCs. The results showed that the administration of this recipient DCs could induce indirect allogeneic-specific cross-hyporesponsiveness to recipient T cells and inhibit the intimal thickening of arterial allograft, which may lead to the suppression of allograft chronic rejection.

## 2. Materials and Methods

### 2.1. Animals

C57BL/6(H-2K^b^), C3H/He(H-2K^k^) and BALB/C(H-2K^d^) male mice weighing 29–32 g, 8–12 weeks old, were purchased from Shanghai Laboratory Animal Center of Chinese Academy of Sciences (Shanghai, China), maintained in a specific pathogen-free facility at Fudan University (Shanghai, China). All animal surgical procedures were approved by the Institutional Animal Care and Use Committee of Fudan University.

### 2.2. Synthesis and Purification of B7AP [26]

Antisense peptides were synthesized and purified by GL Biochem Ltd., (Shanghai). It has been reported that the MYPPPY motif is the core of CD28 binding sites to its ligand B7. Several different peptides containing the motif were screened by BIOPOLYMER and BINDING SITE ANALYSIS in the INSIGHT II molecular modeling software package, and B7AP was obtained with the sequence EFMYPPPYLD. The peptide was synthesized on a solid phase peptide synthesizer (Multiple Peptide Synthesizer; Genemed Synthesis, Inc., CA, USA). The crude peptide was purified by the Varian Prostar high performance liquid chromatography (HPLC) system using a C8 column (Varian Prostar HPLC system, CA, USA). Analytical HPLC was performed through a Varian C8 analytical column using a linear gradient of 0–100% acetonitrile in water containing 0.1% trifluoroacetic acid over a period of 20 min. The identity of the peptide was confirmed by mass spectrometry (Voyager Elite model, Perceptive Bio system, Applied Bio systems, WA, USA). The purity of the peptide (higher than 95%) was examined by HPLC analysis. Peptide was lyophilized and stored at −20°C. Serum-free RPMI 1640 was added to adjust the concentrations of B7AP before use.

### 2.3. Preparation of the Donor Antigen

Freshly harvested C57BL/6 mice spleens were first minced in 2 mL complete RMPI-1640 medium, filtered through a nylon mesh, and then transferred into a 15-mL centrifugal tube. After centrifugation at 1500 rpm for 5 min followed by discarding of the supernatant, complete RPMI-1640 medium was added to the splenocyte suspension. The suspension was then transferred to another centrifugal tube containing 3 mL lymphocyte separation medium and centrifuged at 2000 rpm for 20 min to get the lymphocyte layer. Then the lymphocyte layer was carefully transferred to another centrifugal tube, added with complete RPMI 1640 medium and centrifuged again at 1500 rpm for 5 min. After discarding the supernatant, complete RPMI 1640 medium was added and the cell concentration was adjusted to 1 × 10^6^/mL to get the pure lymphocyte suspension. Lymphocyte suspension (1 × 10^6^/mL) was frozen at −80°C (or liquid nitrogen) and thawed at 37°C for 6 times, then centrifuged at 7500 rpm for 10 min. The supernatant was harvested, filtered through 0.22 *μ*m membrane, and stored at 4°C.

### 2.4. Propagation of Bone-Marrow-Derived DCs Loaded with Donor Antigen

Bone-marrow-derived dendritic cells (BM-DCs) were generated as previously described [27, 28] with some modification. Bone marrow cells harvested from the femurs and tibias of C3H/He mice were cultured in 24-well plates (1 × 10^6^ cells per well). Culture medium contains 160 U/mL gentamycin, 2 mmol/L L-glutamine, 0.05 mmol/L 2-mercaptoethanol, 1 mmol/L sodium pyruvate, and 10% (v/v) FCS (Gibco, Gaithersburg, MD, USA) in the presence of recombinant mouse granulocyte macrophage colony-stimulating factor (rmGM-CSF,10 *μ*g/mL;  R&D Systems, Minneapolis, MN, USA). All cultures were incubated at 37°C with 5% humidified CO_2_. Nonadherent cells would be released spontaneously from proliferating clusters after 48 hours of culture. Medium change containing rmGM-CSF was done every two days. In the sixth day, the donor antigen and recombinant mouse TNF-*α* (rmTNF-*α*, 50 *μ*g/L; R&D Systems, Minneapolis, MN, USA) were added to the medium. In the eighth day, the buoyant cells were harvested at the concentration of 1 × 10^6^ cells/mL. DCs were irradiated with 3000 rads *γ*-ray and then incubated with B7AP (10 mg/L) at 37°C for 90 min. After washing with phosphate buffered saline (PBS) once, tolegenic DCs were modulated at the concentration of 3 × 10^6^/mL. The purity of DC preparations was routinely monitored by flow cytometric analysis using anti-CD11c monoclonal antibody (eBioscience, San Diego, CA, USA). This DC preparation protocol could enrich CD11c + cells more than 85%.

### 2.5. Flow Cytometry

Cell-surface phenotypic analysis of B7AP-treated DCs was done by EPICS ELITE flow cytometer (Coulter, Hialeah, FL, USA). Fluorescein-isothiocyanate- (FITC-) conjugated antimouse MHC Class II, CD80 and CD86 antibody (anti-MHC Class II-FITC, anti-CD80-FITC, anti-CD86-FITC) were used for cell staining (BD PharMingen, San Diego, CA, USA). FITC-conjugated isotype-matched irrelevant monoclonal antibodies (rat IgG2b, Armenian hamster IgG2, and rat IgG2a, resp.) were used as negative controls (Cedarlane Laboratories, Hornby Ontario, Canada).

### 2.6. Mixed Lymphocyte Reaction (MLR)

T cells prepared from recipient splenocytes by nylon wool purification were used as responders (2.5 × 10^6^/mL). B7AP-treated recipient DCs (0.5 × 10^6^ from C3H/He mice) were used as stimulators and irradiated with 3000 rads before use. Untreated recipient DCs pulsed with donor antigen and recipient DCs without donor antigen were included as controls. B7AP was diluted to 4 × 10^2^ mg/L, 4 × 10^1^ mg/L, 4 × 10^0^ mg/L,4 × 10^−1^ mg/L, 4 × 10^−2^ mg/L, 4 × 10^−3^ mg/L, 4 × 10^−4^ mg/L, and 4 × 10^−5^ mg/L by serum-free RPMI 1640 and then incubated with recipient DCs (0.05 mL DCs and 0.05 mL B7AP) for 1 hour at 37°C. After that, MLR between DCs and T cells was performed. Cultures were established in triplicate in 96-well round-bottom microculture plates (200 *μ*L per well) and maintained in complete medium for 7 days in 5% CO_2_ at 37°C. ^3^[H] TdR (1 *μ*Ci/well) was added in the final 18 hrs. Cells were harvested onto glass fiber disks using an automated system, and incorporation of  ^3^[H] TdR into DNA was assessed by Wallac 1450 Microbeta liquid scintillation counter. Data was presented as mean counts per minute (cpm) ±SD in triplicate replication.

### 2.7. Induction of Indirect Allogeneic-Specific Hyporesponse to Recipient T cells *In Vitro*

Mixed lymphocyte reaction (MLR) between recipient T cells and B7AP-pretreated recipient DCs pulsed with donor antigen was performed in complete medium for 72 hrs in 5% CO_2_ at 37°C. Then PBS was added and the suspension was harvested by centrifugalization at low rotation speed (500 r/m  × 15 min) to remove the precipitated dead cells. After being washed for three times by PBS, the pretreated T cells were adjusted at the concentration of 5 × 10^6^/mL and placed in static condition for 24 hrs. For the second round of MLR, the pretreated T cells were used as responders (2.5 × 10^5^ per well). The recipient DC pulsed with donor antigen untreated by B7AP was used as stimulators (0.5 × 10^5^ per well). The recipient DC pulsed with the third party donor (derived from BALB/C) without B7AP treatment and the recipient DC without donor antigen were included as controls. Cell harvesting and thymidine incorporation was performed the same way as the first round of MLR.

### 2.8. Murine Model of Allogeneic Carotid Artery Orthotopic Transplantation

Both C3H/He-recipient mice and C57BL/6 donor mice were anesthetized with ketamine (75 mg/kg, i.p.), had their hair shaved, and then placed in a supine position with their limbs immobilized. The skin of the operative region was sterilized. Longitudinal incisions from the right mandibular angle to the middle point of the right clavicle were made for both donor and recipient surgeries. Common carotid artery of the donor was dissected, excised for a 0.5 cm segment, and then flushed and stored at 4°C Ringer's solution. After the dissection and removal of a 0.5 cm segment of recipient carotid artery, the arterial graft was anastomosed to the recipient carotid artery orthotopically using an end-to-end interrupted suture technique with 10-0 PROLENE at 40x magnification. The distal end of the carotid was first reperfused, followed by the reperfusion of the proximal end under the condition of no anastomotic bleeding. The incision was closed with layered sutures. Animals were allowed access to food and water right after surgery. 

To assess the effect of the pretreated DCs on the intimal hyperplasia of the arterial graft, recipient mice (*n* = 10) were given 3 × 10^6^ cells intravenously via the femoral vein, 7 days before transplantation in the absence of immunosuppression. Meanwhile, control group (*n* = 10) were established without any treatment. Arterial grafts were harvested for histopathological examination 2 months after transplantation.

### 2.9. Morphometric and Histological Analysis

Cross sections of arterial grafts were fixed in 10% formaldehyde and then embedded into paraffin and stained with hematoxylin and eosin. The area of the tunica intima and tunica media was assessed by light microscopy and computer-based Morphometric Analysis System. Only vessels that were cut orthogonally and displayed a clear internal and outer elastic lamina were accepted. The thickness of tunica intima was calculated by the following equation: Thickness of tunica intima = Area of tunica intima/(Area of tunica intima + Area of tunica media) [29].

### 2.10. Statistical Analysis

All data are presented as means ± SD. Statistical analysis was performed by STATA version 8.0. Homoscedasticity analysis and *t* test were used to analyze the significance of differences between groups for the proliferative response of T cells and the thickness of the arterial intima. *P* value less than 0.05 was considered as statistically significant.

## 3. Results

### 3.1. Propagation of Murine Recipient BM-DCs Loaded with Donor Antigen

**Figure 1:**
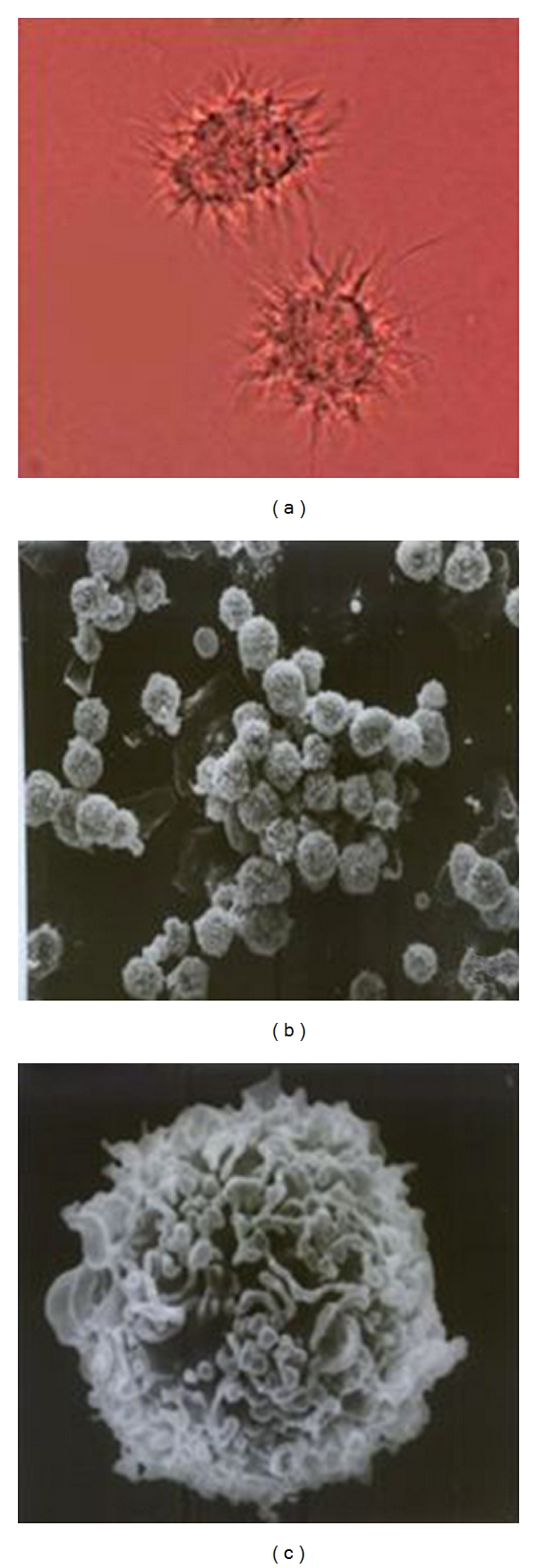
BM-DCs detected by inverted microscope and scanning electron microscope. Progenitor cells were propagated in the presence of rmGM-CSF. After TNF-*α* and donor antigen addition, the cells show significant differentiation with typical DC appearance under inverted microscope (a) and scanning electron microscope (b) and (c).

**Figure 2:**
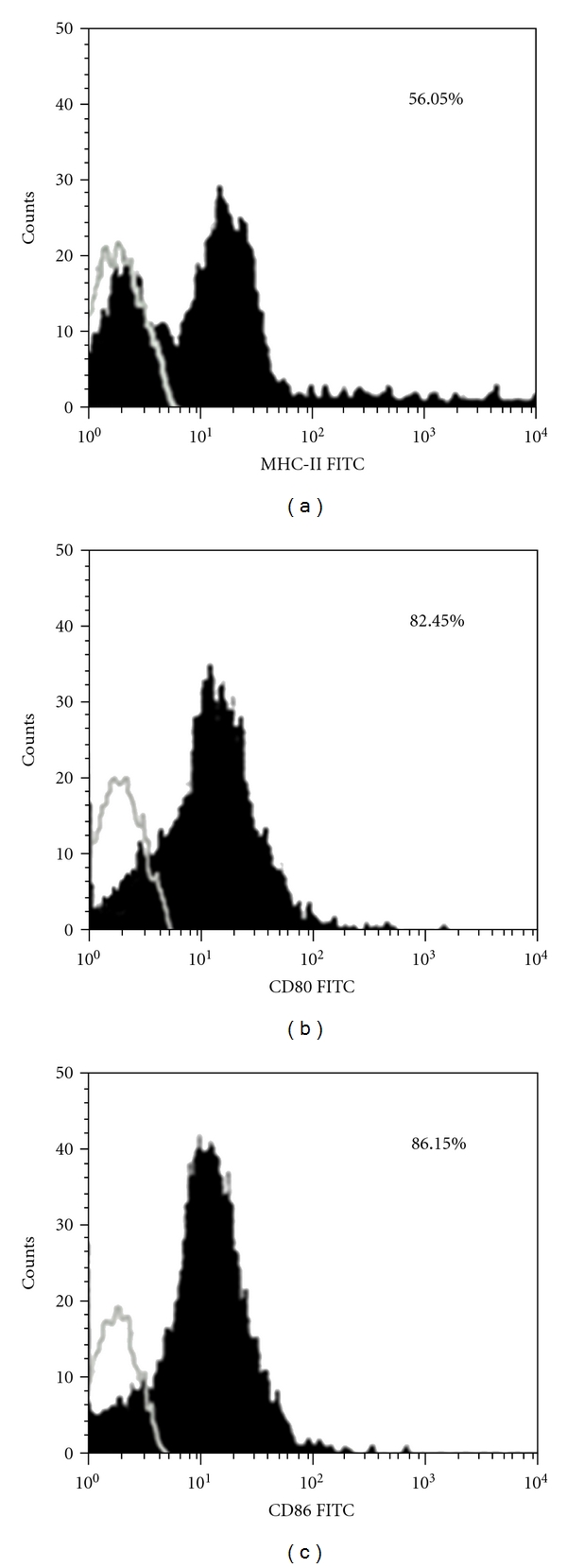
Expression of MHC-II (I-Ak) and B7 molecules (CD80/86) on BM-DCs. The expression of B7 or MHC-II by the indicated cell fractions is represented by filled histograms (black). The open histograms (grey) represent control staining with an isotype control antibody. Flow cytometric analysis demonstrated that over 55% of the cells express MHC-II (I-Ak) molecule (a), while 80 + % of the cells expressed B7.1 (CD80) and B7.2 (CD86) molecules (b) and (c). One representative of three independent experiments is shown.

A large number of progenitor cells were propagated in the presence of rmGM-CSF. After TNF-*α* was added in the medium, the cells show significant differentiation with typical DC appearance [30] (Figure 1) under inverted microscope and scanning electron microscope. Majority of the progenitor cells differentiated to DCs as MHC-II (I-Ak) molecule are expressed on 55 + % of the cells by flow cytometric analysis and 80 + % of the cells expressed B7(CD80,CD86) molecules (Figure 2). 

### 3.2. Inhibition of T-cell Proliferative Response Using B7AP Pretreated DCs

**Figure 3:**
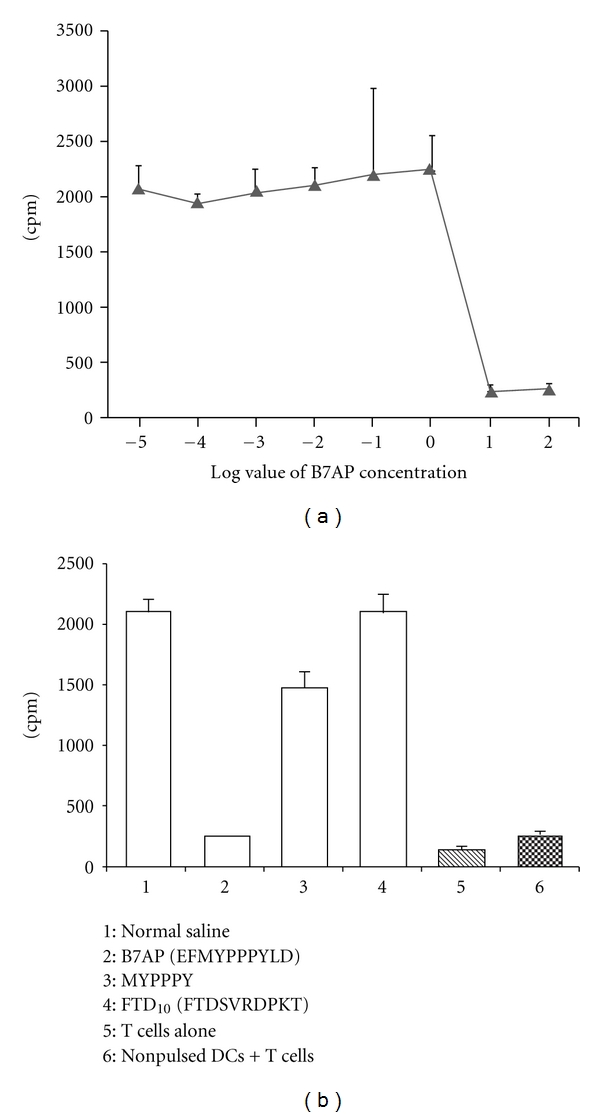
B7AP-pretreated DCs inhibited T-cell proliferation in MLR. Under high concentrations of B7AP (100 mg/L), T cells demonstrated a low proliferation capacity. However, proliferation of T cells surged to a rather high level when the final concentration of B7AP went down from 10 mg/L to 1 mg/L, followed by a plateau (a), indicating that B7AP final concentration of 10 mg/L could ensure maximal blockage of DC surface B7 molecules. The BM-DCs were pretreated by the B7AP and control peptide at the same concentration (10 mg/L), and the MLR was performed as described above (b). NS: normal saline group, MYPPPY group: relative peptide, FTD10 (FTDSVRDPKT) group: negative control peptide.

In order to find the concentration of B7AP that can maximally block B7 molecules, DCs were incubated with B7AP at various concentrations, and then MLR between DCs and T cells was performed. B7AP could inhibit the proliferative response of T-cells compared with untreated group (*P* < 0.05, Table 1) and perform the maximal blockage at the end-point concentration of 10 mg/L (Figure 3(a)). Furthermore, to test the specificity of the inhibitory effect of B7AP, a control peptide (FTD_10_) was synthesized and the MLR was performed at the same concentration of B7AP (10 mg/L). A relative peptide, MYPPPY, was also tested for its inhibitory effect in the MLR at the concentration of 10 mg/L. 

As shown in Figure 3(b), all groups have significant differences compared to the B7AP group (*P* < 0.05). The FTD_10_ control group showed no significant difference compared to the normal saline (NS) group (2087 ± 150 versus 2102 ± 101, *P* > 0.05). The relative peptide MYPPPY showed a certain extent of inhibitory effect, but also with no significant difference compared to the NS group (1480 ± 130 versus 2087 ± 150, *P* > 0.05). The data demonstrated that the inhibitory effect of B7AP was specific (Figure 3(b)).

### 3.3. Induction of Alloantigen-Specific T-cell Hyporesponsiveness Using B7AP Pretreated DCs

The proliferative response of T cells was examined in secondary MLR, which has shown that T cells harvested from the primary MLR exhibited markedly reduced responses to alloantigen (C57BL/6) versus the third party unrelated antigen (BALB/C), indicating B7AP pretreated DCs could induce alloantigen-specific T-cell hyporesponsiveness. (*P* < 0.05, Table 2).

### 3.4. Inhibition of Arterial Allograft Intimal Hyperplasia

**Figure 4:**
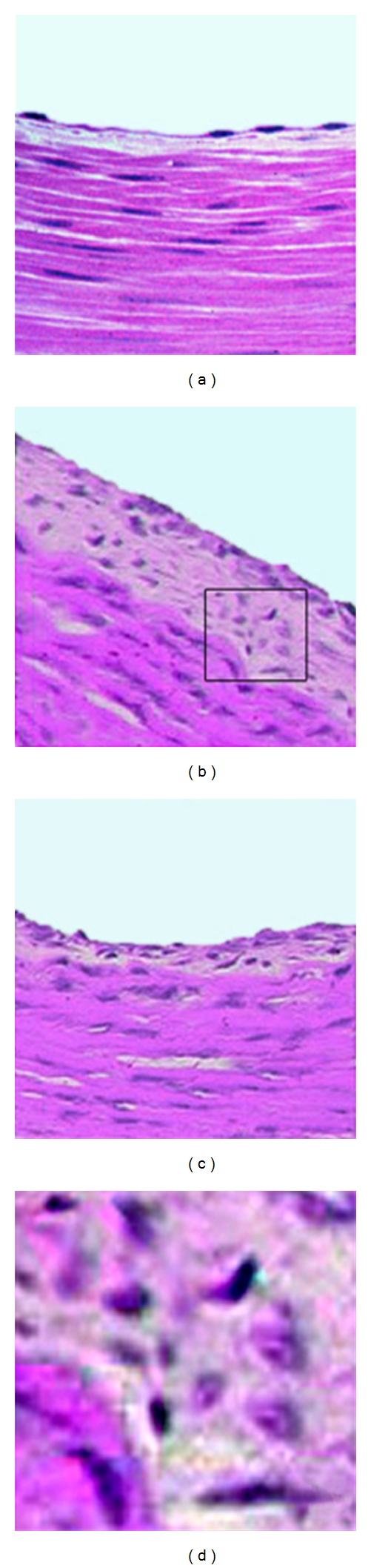
HE stained sections of the arterial grafts 2 months after transplant. (a) native carotid artery; (b) carotid arterial allograft (untreated control); (c) carotid arterial allograft (pretreated); (d) zoomed-in picture of the square fragment in Figure 4(b). Grafts in both DC-pretreated and control group showed diffused and concentric intimal thickening compared with normal nontransplanted artery (a), which is the main feature of chronic allograft rejection. However, the intima was significantly thinner in the pretreated group (c) than that in the control group (b). Also we could see more lymphocytes infiltrated within control group allografts (d).

A total of 20 transplants were performed with the surgical successful rate of 100%. All mice and arterial grafts survived 2 months. The arterial grafts were examined under microscope 2 months after transplantation, and three histologic sections of every allograft were analyzed. During specimen harvesting, arterial grafts in the pretreated group presented stronger pulse compared to the control group. In all arterial grafts, histopathological examination demonstrated diffused and concentric intimal thickening compared to the normal nontransplanted artery, which involves the entire circumference of the vessel with a chronic picture at 2 month. However, the mean intimal thickness was significantly reduced in the pretreated group compared to the control group (mean intimal thickness: 0.071 ± 0.03  versus 0.179 ± 0.056, *P* < 0.05, Figure 4).

## 4. Discussion

In the field of organ transplantation, it is accepted that both donor and recipient DCs mediate the allograft rejection. The recognition of foreign major histocompatibility complex (MHC) molecules by recipient alloreactive T-cells via two distinct pathways, “direct” (donor DCs presenting donor MHC molecules) and “indirect” (recipient DCs presenting donor MHC molecules), is one of the major causes of different types of organ transplant rejection [2, 3]. It has been suggested that the direct pathway predominates during early acute rejection and the indirect pathway provides a continuous supply of alloantigen responsible for chronic rejection later [4, 5]. Animal trials found that blocking the direct allorecognition pathway only did not attain allograft tolerance, which indicates that maintaining long-term clinical transplantation tolerance by arresting the indirect pathway is essential [3]. In addition, administration of donor-derived dendritic cells (DCs) to prevent allograft rejection is not applicable for clinical use. We therefore attempted to explore the use of recipient-derived DCs pulsed with donor antigens via the indirect pathway.

T-cell activation is a process involving alloantigen recognition and costimulatory signaling. Besides the interaction of the TCR and MHC-antigen complex, a productive immune response and maintenance of T-cell homeostasis are determined largely by co-stimulatory molecules [31]. Co-stimulatory molecule-deficient DCs have the capacity to control immune responses and induce T anergy [25, 32]. It would result in the alloantigen-specific T hyporesponse. The most active pathway of costimulation is the interaction of CD28 receptor and B7 ligands [7].

Aortic allotransplantation in mice is a useful experimental model to study the mechanisms of chronic rejection in allotransplantation [33–35]. However, the application of the conventional aortic model is limited by a high morbidity and technically difficult to perform. So we developed a new simple method of carotid artery orthotopic allotransplantation in mice. This new procedure is easy to carry out and has a low morbidity after extensive training based on our experience.

Compelling evidence that induction of tolerance in the indirect pathway favors graft survival came from experiments in which blockade of CD28-B7 by monoclonal antibodies (MoAbs) against B7 or CTLA4-Ig could produce indefinite allograft survival [36]. Antisense peptides are short peptide sequences that specifically constitute one side of the binding sites of complementary protein pairs [24]. B7AP is a peptide analogue of the CD28-binding region [26, 37], which can be characterized by higher affinity to B7 ligand, lower molecular weight, and lower immunogenicity and difficult to be metabolized compared with CTLA4-Ig [26, 38]. It would block the allorecognition in a longer period to take more effect. Chen et al. reported [26] that blockade of B7 molecules on donor splenocytes with B7AP could induce specific immune hyporesponse and prolong allograft survival in the recipients. Our study also confirmed that B7AP could inhibit the T-cell proliferative response stimulated by DCs. Transient blockade of B7-CD28 (using CTLA4Ig) did not abrogate the development of the intimal hyperplasia [3, 39]. It was uncertain whether more stable blockade of the indirect allorecognition would be more effective to inhibit intimal thickening in carotid artery allotransplantation. Using B7AP can lead to a stable blockade of CD28-B7 costimulation by virtue of its characters. The result found recipient BM-DCs pulsed with donor antigen and pretreated by B7AP could induce recipient T-cell-specific hyporesponse to the donor antigen *in vitro* and significantly alleviate the intimal thickening in the murine allotransplantation, which is a key manifestation of chronic rejection. It indicates that an approach to use recipient DCs as a “vaccine” strategy provides a feasible approach to inhibit the chronic rejection in organ transplantation.

## 5. Conclusion

Our research has demonstrated that blockade of B7/CD28 costimulatory pathway by B7AP in the indirect allorecognition could induce allogeneic-specific cross-hyporesponsiveness and inhibit the arterial allograft intimal hyperplasia due to chronic rejection, which may be involved in future strategies for human allograft chronic rejection.

## Figures and Tables

**Table 1:** MLR of T cells and recipient DCs with different treatment.

Groups	Final concentration of B7AP	Cpm
B7AP-pretreated ^C57BL/6a^C3H/HeDCs + T cells		

Subgroup 1	100 mg/L	252 ± 40
Subgroup 2	10 mg/L	236 ± 36
Subgroup 3	1 mg/L	2239 ± 316
Subgroup 4	1 × 10^−1^ mg/L	2197 ± 777
Subgroup 5	1 × 10^−2^ mg/L	2097 ± 150
Subgroup 6	1 × 10^−3^ mg/L	2039 ± 210
Subgroup 7	1 × 10^−4^ mg/L	1935 ± 79
Subgroup 8	1 × 10^−5^ mg/L	2070 ± 208

Untreated ^C57BL/6^C3H/HeDCs + T cells	0	2106 ± 326
DCs without donor antigen + T cells	0	252 ± 125
T cells alone	0	124 ± 35

**Table 2:** MLR of pretreated T cells and recipient DCs pulsed with different donor antigen.

Groups	Cpm values
Pretreated T cells + ^C57BL/6^C3H/He DC	355 ± 46
Pretreated T cells + ^BALB/C^C3H/He DC	2230 ± 248
Control	105 ± 22

^C57BL/6^C3H/HeDC: Recipient DCs pulsed with donor antigen (derived from C57BL/6).

^BALB/6^C3H/HeDC: Recipient DCs pulsed with the third party antigen (derived from BALB/C).
